# Exploring the dynamics of messenger ribonucleoprotein-mediated translation repression

**DOI:** 10.1042/BST20231240

**Published:** 2024-11-27

**Authors:** Julia Meyer, Marco Payr, Olivier Duss, Janosch Hennig

**Affiliations:** 1 Department of Biochemistry IV – Biophysical Chemistry, University of Bayreuth, 95447 Bayreuth, Germany; 2 Molecular Systems Biology Unit, European Molecular Biology Laboratory (EMBL), 69117 Heidelberg, Germany; 3 Candidate for Joint PhD Degree From EMBL and Heidelberg University, Faculty of Biosciences, Heidelberg, Germany

**Keywords:** RNP dynamics, RNP structure, single-molecule fluorescence microscopy, translation regulation

## Abstract

Translational control is crucial for well-balanced cellular function and viability of organisms. Different mechanisms have evolved to up- and down-regulate protein synthesis, including 3′ untranslated region (UTR)-mediated translation repression. RNA binding proteins or microRNAs interact with regulatory sequence elements located in the 3′ UTR and interfere most often with the rate-limiting initiation step of translation. Dysregulation of post-transcriptional gene expression leads to various kinds of diseases, emphasizing the significance of understanding the mechanisms of these processes. So far, only limited mechanistic details about kinetics and dynamics of translation regulation are understood. This mini-review focuses on 3′ UTR-mediated translational regulation mechanisms and demonstrates the potential of using single-molecule fluorescence-microscopy for kinetic and dynamic studies of translation regulation *in vivo* and *in vitro*.

## Introduction

Maintaining life requires a great amount of energy, whereby organismal processes consume different quantities of energy carriers. To avoid wasting energy unnecessarily, processes in the cell must be highly regulated. The most energy-consuming process is protein synthesis [1]. Regulation of translation is therefore essential to ensure well-balanced cellular functions while using as little energy as possible. During stress responses or developmental steps like oogenesis, spermatogenesis and early embryonic development, where DNA is transcriptionally silenced, proteins need to be synthesized in a short period of time [2,3]. Here, the cell is dependent on translational control, as mRNAs are highly abundant in the cytoplasm and their translation needs to be up- or down-regulated. Failure of these control mechanisms is often linked to disease [4,5]. Therefore, a detailed molecular deciphering is needed to understand and potentially counteract disease pathways. Despite the availability of numerous high-resolution structures of the ribosome and its associated complexes during all stages of translation [6–13], the mechanisms that regulate translation initiation on the level of 43S pre-initiation complex (PIC) recruitment remain elusive. We have a lack of understanding on the detailed interactions of PIC components with general RNA binding proteins (RBPs) and how these interactions regulate the process of PIC recruitment to the mRNA. Also, the kinetics and dynamics of RBP-binding to untranslated regions (UTRs) of the mRNA and their interactions with the PIC are less understood. For example, it is not clear how RBPs modulate 43S PIC recruitment by specifically interacting with the UTRs to efficiently inhibit translation initiation which occurs for instance during repression of dosage compensation in female *Drosophila melanogaster* (see below). Regarding translation activation, recent studies have revealed the involvement of PIWI proteins and Piwi-interacting RNAs in translation up-regulation of mRNAs in developmental processes in *Drosophila* and mammals, whereas detailed mechanistic insights are unknown yet as well [14,15]. Thus, translation regulation proves to be critical in various different cellular processes, emphasizing the need to understand repression and activation of translation in a spatiotemporal manner. A prominent example is the active transport of translationally repressed *oskar* mRNA as solid-like RNP granules to the posterior pole of the *Drosophila* embryo during embryogenesis. The subcellular localization of the messenger ribonucleoprotein (mRNP) then triggers translation activation necessary for correct abdomen and germline formation [16–19].

In this mini-review, after describing the basics of translation initiation, we focus on summarizing the mechanistic understanding of regulation of eukaryotic translation repression. We then outline how *in vitro* and *in vivo* single-molecule fluorescence imaging methods can be used to study the assembly kinetics of mRNP complexes associated with translation repression thereby providing a holistic understanding of post-transcriptional regulation of gene expression. We refer to more extended reviews, which cover other aspects of translation regulation mechanisms, for example, by RNA modifications or activating long non-coding RNAs [2–5].

## Translation initiation

**Figure 1.**
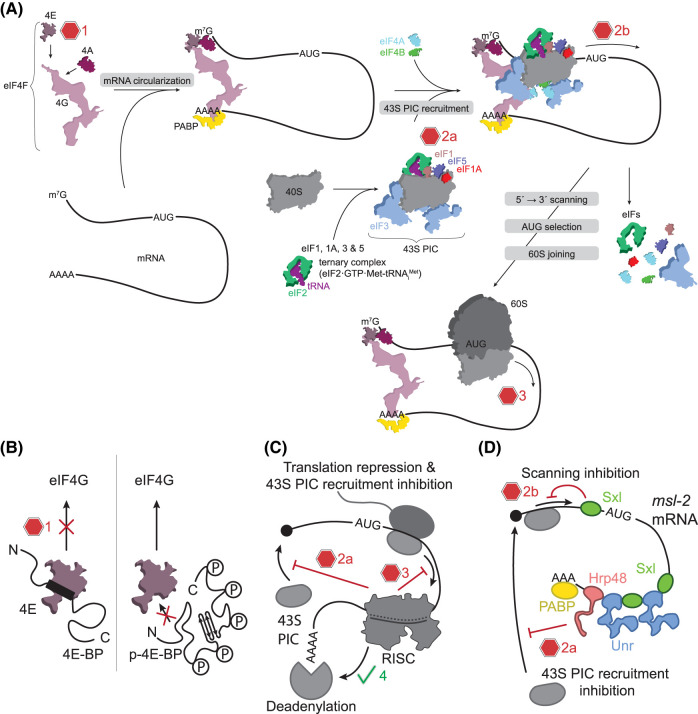
Translation initiation and repression mechanisms. (**A**) In a canonical cap-dependent translation pathway, the cap-binding complex eIF4F activates the mRNA by invoking a 5′-3′ end proximity. In cap-dependent translation initiation, the 43S PIC consisting of the small ribosomal subunit and eIF3/1/1A binds to the 5′ m^7^G cap. This is followed by (1) scanning of the 5′ UTR until (2) the initiation codon is recognized and (3) 60S subunit joining. This primes the ribosome for an elongation competent state. (**B**) Translation repression is mediated through 4E-BP which masks the eIF4G binding site on eIF4E by interacting with the eIF4G binding interface on eIF4E depending on the phosphorylation state of 4E-BP. A stable association of the PIC is thereby inhibited. (**C**) Translation repression via miRISC-mediated gene silencing occurs in a multitude of steps in which 43S PIC recruitment can be targeted (2a), slow-down of translation (3) and/or deadenylation and subsequent mRNA decay can be promoted (4). (**D**) The engagement of the 43S PIC is inhibited through the 3′ UTR of *msl-*2 mRNA and *trans*-acting RBPs such as Sxl, Unr, Hrp48 and PABP (2a). As a fail-safe mechanism, leaky recruitment is counteracted by Sxl binding to the 5′ UTR, which prevents translation via interference with the scanning mechanism (2b).

Eukaryotic translation can be divided into three main processes: initiation, elongation, and termination. Translation initiation is the rate-limiting step of protein synthesis and the main target for regulatory RBPs [2]. It has been shown that translation initiation in eukaryotes is a highly organized multi-step process in which several different eukaryotic initiation factors (eIFs), transfer RNA (tRNA), and, coupled to GTP hydrolysis, the ribosomal subunits assemble on the mRNA. First, the ternary complex (TC), consisting of the initiator tRNA (Met-tRNA_i_) and GTP-bound eIF2, associates with eIF5 and the small 40S ribosomal subunit, which is already in complex with eIF3/1/1A (Figure 1A) [20–23]. This 43S PIC is recruited to the 5′ m^7^G-cap of the circularized mRNA and interacts via eIF3 with the cap-binding complex eIF4F. The cyclization of the mRNA, established through interactions between eIF4G and the poly(A) binding protein (PABP), promotes translation re-initiation of terminating ribosomes that dissociate from the mRNA at the 3′ end by bringing both termini of the mRNA into close spatial proximity [24]. To detect the initiation codon, the 43S PIC scans the mRNA in the 5′-3′ direction for the AUG motif in an ATP-dependent manner [3,25–27]. During this process, eIF5 promotes GTP-hydrolysis of the eIF2·GTP complex, whereby the γ-phosphate stays located in the P-site of the 43S PIC [28]. The release of the γ-phosphate and dissociation of eIF2·GDP is triggered upon AUG-recognition and stabilizes the closed accommodated (*P*_in_) conformation in which the Met-tRNA_i_ stably binds to the start codon [27,29]. Release of the γ-phosphate acts as a commitment step [29]. In a final step, eIF5B mediates joining of the 60S ribosomal subunit with the newly formed 48S initiation complex (mRNA·eI4F·43S PIC) and the elongation competent 80S ribosome is assembled [13,27]. Although the process of translation initiation is well-studied, kinetic aspects of the single events and the determination of rate-limiting steps within translation initiation remain partially elusive and need further investigation (PMID: 38052923; PMID: 32883864; PMID: 38287194). However, first observations of 43S and 48S initiation complexes using sucrose gradient analyses suggest, that more than two slow steps can occur during initiation such as mRNA binding of the 43S PIC and, concerning the 48S PIC, the scanning process or the subsequent subunit joining [30]. Kinetic studies on translation initiation revealed that, while scanning is very rapid, 43S PIC recruitment and 60S ribosome joining are comparably slower, whereby eIF1A and eIF5B help to facilitate 60S joining for this commitment step [31–33]. In contrast, a mechanistic and kinetic understanding of the steps necessary for 43S PIC recruitment and how it can be modulated by regulatory elements is incomplete and will be of future importance.

In addition to the aforementioned cap-dependent translation initiation, alternative mechanisms have evolved and are active, for example, in cases of cellular stress or viral infection [34,35]. Here, eIFs are recruited to internal ribosomal entry sites located at the 5′ UTR, that allow assembly of the ribosome despite the absence of an m^7^G-cap structure [35,36]. However, this mini-review focuses explicitly on translation repression mechanisms concerning the canonical cap-dependent initiation.

## Translation repression by trans-regulatory factors

Global and specific control of post-transcriptional gene expression can be targeted at many stages throughout the translation cycle. Translational control is especially important during translation initiation as the rate-limiting step of translation [37–40] (reviewed in [41]). Many different translation repression mechanisms exist in eukaryotes that regulate either global protein levels in the cell or control the expression of specific mRNAs. Global regulation usually occurs upon cellular stress responses such as nutrition deprivation, viral infection or endoplasmic reticulum stress [42]. Here, different protein kinases (depending on the type of cellular stress) phosphorylate the α-subunit of eIF2 which inhibits the GDP-GTP exchange for re-activation of the TC [42,43]. Another regulation mechanism for global protein expression is triggered by the mTORC signaling pathway. The protein kinase mTORC1 is activated through extracellular signals and phosphorylates eIF4E-binding proteins (4E-BPs) which inhibit cap-dependent translation initiation [43]. The latter mechanism is also exploited in regulation of specific mRNAs which will be tackled in the following section in which we also focus on 3′ UTR mediated translation repression.

In general, the 3′ UTR can accommodate regulatory sequence and/or structure elements that can act as a binding platform for regulatory RBPs. One well-known 3′ UTR-mediated translation repression mechanism during the initiation step is the competition of 4E-BPs with eIF4G for eIF4E (Figure 1B) [44–47]. 4E-BPs can be recruited by RBPs to the 3′ UTR to interfere with the association of eIF4E with eIF4G. The stable association of the 43S PIC with the 5′ cap is usually established through eIF4G and is therefore prevented via 3′ UTR bound 4E-BPs [46]. A prominent example for 4E-BPs in *D. melanogaster* is Cup which represses translation during oogenesis and early embryonic development [48–50]. During determination of the fruit fly's body axes, Cup is involved in repression of multiple mRNAs, such as *oskar* [48], *gurken* [51], *nanos* [52], where it is recruited to the 3′ UTR by different mRNA-specific repressor proteins and binds eIF4E, thus preventing eIF4E-eIF4G interactions [49,50,52,53]. Structural analysis of the eIF4E-Cup complex provided insights into eIF4E-mediated repression mechanisms [49]. Cup harbors two α-helical eIF4E-binding modules at its N-terminus: A canonical binding motif (4E-BS I) and a non-canonical binding site (4E-BS II) [49]. 4E-BS I interacts with eIF4E similar to eIF4G and other 4E-BPs which makes binding of Cup and eIF4G to eIF4E mutually exclusive. Next to eIF4G replacement, Cup has a second function which is putatively caused by 4E-BS II. Although 4E-BS II has lower binding affinity to eIF4E, it increases the affinity of eIF4E for the 5′ cap which finally stabilizes and protects the mRNA from decapping and degradation events [49]. Other 4E-BPs derived from vertebrates (4E-BP1-3), fly (Thor) or yeast also comprise the canonical 4E-BS I, whereas the α-helical 4E-BS II structure is a unique feature of Cup [54]. The affinity is fine-tuned by phosphorylation of 4E-BP1-3, and involves a conformational change of 4E-BPs upon phosphorylation [55]. The phosphorylation of 4E-BPs is mediated by the mTORC1 pathway and interferes with the interaction of eIF4E and 4E-BPs (Figure 1B) and dysregulation of this pathway leads to many diseases linked to up-regulation of translation such as cancer [56], obesity and diabetes [57–59] as well as neurological disorders [60]. Excessive protein synthesis in brain cells, for example, can cause neurological dysfunctions resulting in autism-like disorder (ASD) and fragile X syndrome, the latter leading to hyperactivity, deficiencies in learning and memory capabilities [61]. This neurological disease is triggered by loss of function mutations within the *FMR1* gene [62]. Its gene product, FMRP, is an RBP that enhances translation of 4E-BP2, a neuron specific homologue of 4E-BP1 which is responsible for regulation of synaptic function via cap-dependent translation repression [63]. Recent findings investigating specifically inhibitory and excitatory neurons as well as astrocytes showed that loss of 4E-BP2 in inhibitory neurons in mice causes autism-like behavior [64]. These results confine the location that is most affected upon loss of 4E-BP2 activity and provide further information for future treatment approaches of ASD.

Another well-studied translation repression mechanism, which acts on a specific set of mRNAs, is microRNA (miRNA)-dependent translation repression. Here, miRNAs associated with Argonaute (Ago) proteins bind to the 3′ UTRs of specific eukaryotic mRNAs, that contain miRNA binding sites. Binding of miRNAs can interfere with various steps of translation initiation and act also during translation elongation through ribosomal stalling [41,65]. All four human Ago paralogs recognize miRNAs [66]. The miRNA-induced silencing complex (miRISC) comprises miRNA complexed by an Ago protein, which comprises four domains: the N-terminal domain (N), the Piwi–Argonaute–Zwille (PAZ) domain, the middle (MID) domain and the P-element induced wimpy testes (PIWI) domain [67]. Nucleotide 1 of the miRNA (5′ end) is recognized by Ago and nt 2–8 serve as a seed region to screen for target sites on the mRNAs. The 3′ supplementary region of the miRNA can interact with the PAZ domain to facilitate target site recognition [68]. Altogether, RISC-mediated gene silencing can act on multiple different hierarchical levels of translation such as activated deadenylation and subsequent RNA decay, slow-down of translation elongation or inhibition of initiation, for which a mechanistic understanding is incomplete [68,69] (Figure 1C).

Another step in translation initiation, which is highly regulated, is the recruitment of the 43S PIC (Figure 1A,D). Inhibition of 43S PIC recruitment can be achieved through direct interactions of repressor proteins with the PIC. A well-known example for this repression mechanism is suppression of dosage compensation in female *D. melanogaster*. Here, the assembly of the dosage compensation complex, that triggers hypertranscription in male flies, is prevented through translational silencing of *male-specific lethal 2* (*msl-2*) mRNA which is the rate-limiting component for dosage compensation complex formation [71]. Sex-lethal (Sxl), which is the main RBP to repress translation of the *msl-2* mRNA, is exclusively expressed in female flies and binds specifically to poly(U) stretches within the 5′ and 3′ UTR [72]. Together with the RBPs PABP, Hrp48, and Unr, which binds cooperatively with Sxl to the 3′ UTR of *msl-2* mRNA, this set of RBPs inhibits 43S PIC recruitment supposedly via direct contacts of Hrp48 with 43S PIC subunit eIF3d (Figure 1D) [73–75]. Interfering with this mechanism has lethal consequences for female flies [76]. Sxl harbors a second repression mechanism by binding to the 5′ UTR of *msl-2* and interfering with the ribosomal scanning for the start codon, in cases where PICs could escape 3′ UTR-mediated regulation (Figure 1D) [77]. Unr has a highly conserved human counterpart, which is abundantly expressed in several cancer cell lines [78–81]. Unr residues interacting with Sxl in *Drosophila* upon binding to mRNA are also fully conserved [82], however, the Sxl counterpart in humans has not been identified yet. Thus, the *Drosophila* system needs to be further analyzed in the meanwhile to fully understand translation regulation at the level of 43S PIC recruitment. This system is already well-understood on the cellular level, although the possibility remains that other RBPs are involved in translation repression of *msl-2*. Furthermore, knowledge on assembly and mechanisms of action of this repression complex are limited to isolated or smaller parts of complexed components [74,82–84]. Despite providing tremendous insights into novel RNA recognition modes utilized by RBPs [85,86] and confirming direct interactions between Unr and PABP [75,84], the roles of Unr's cold shock domains 2–9 or of Unr-PABP interactions during translation repression remain unclear.

In addition to regulatory RBPs, another important aspect to study is the interplay between non-coding RNAs and the translation machinery. A prominent example are G4-RNA quadruplexes (rG4s), a non-canonical RNA secondary structure element, which can regulate translation initiation [87]. RNA helicases such as DHX36/Rhau, a DEAH-box family RNA/DNA helicase, can specifically recognize these elements and resolve them to modulate translation [88,89]. In general, resolving rG4s can have a variety of effects such as up- and down-regulation of translation as well as affecting mRNA stability, processes which are linked to diseases related to heart development, leukemia or ALS [90–93].

To obtain a detailed mechanistic understanding of these complex translation repression processes, classical structural biology methods, such as X-ray crystallography, NMR and cryo-EM, have and will provide important and detailed insights into the structural basis of repression complexes. However, disseminating mRNP complex assembly and regulation dynamics need complementary approaches, that can look at dynamic processes in a time-resolved manner at physiological concentration ranges.

## Single-molecule methods for studying translation regulation

Investigating the order, stoichiometry, and kinetics of assembly as well as the stability of possible repressive mRNP complexes and translation initiation intermediates is important to obtain mechanistic information about these repression processes. This information is essential for understanding disease pathways related to defective translation repression and subsequently required for effective and successful drug development. While bulk experiments only provide ensemble-averaged information on the biological process under investigation, single-molecule experiments retain the information provided by the heterogenous set of individual molecules. Single-molecule observation allows the detection of rare and transient states and allows following the kinetics of multi-step processes in real-time even if multiple pathways exist in the dynamic molecular ensemble. This is crucial to identify rate-limiting steps that could serve as entry points for medical interventions to specifically block translation initiation. In the following, we will focus on the importance of understanding the assembly and molecular kinetics of mRNP complexes involved in translation repression. We would like to showcase how single-molecule methods *in vitro* and *in vivo* can be used to understand the mechanisms of translation repression in greater detail.

## *In vitro* single-molecule fluorescence microscopy

While experiments *in vivo* complicate dissecting direct from indirect effects upon cellular manipulation, *in vitro* reconstitutions allow a precise control of components and experimental conditions. A powerful method to study the dynamics of single molecules *in vitro* is single-molecule total internal reflection fluorescence (TIRF) microscopy (see Box 1). This method allows us to study the dynamics of molecules immobilized to a functionalized glass surface and to track the binding of fluorescently labeled ligands to the immobilized molecules in real-time.

Box 1. **Single-molecule TIRF**

**Figure 2.**
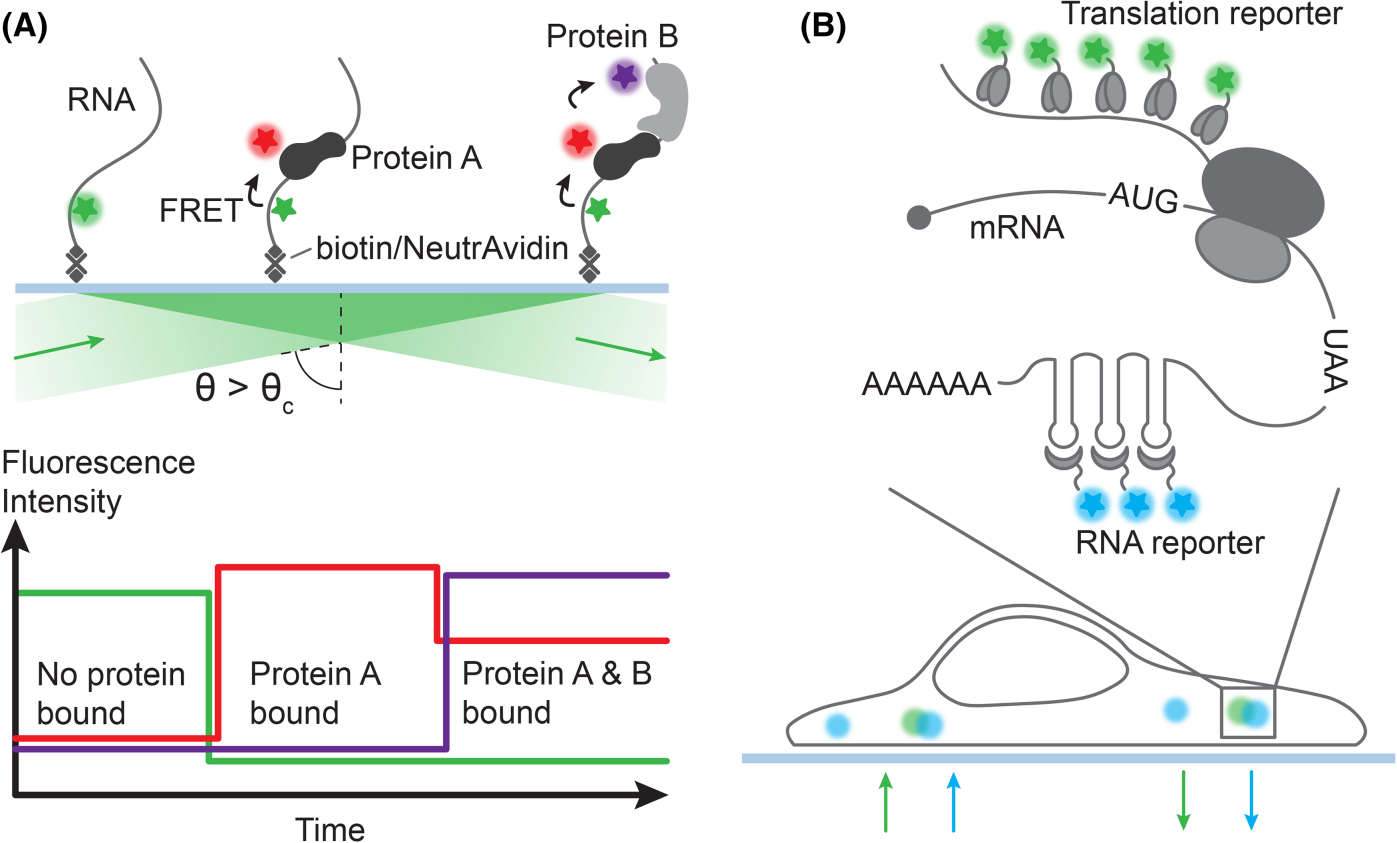
Schematics of multi-color single-molecule fluorescence microscopy experiments *in vitro* and *in vivo*. (**A**) *In vitro*: in a single-molecule TIRF microscope set-up, the excitation beam travels above a critical angle *θ_c_* through the glass coverslip and gets totally reflected. Thereby, an evanescent field parallel to the coverslip is created which excites the FRET donor fluorophore (green) located close to the surface. This allows monitoring immobilized molecules (for example RNA) labeled with a FRET donor dye (green) over time. In an *in vitro* set-up, protein A conjugated to a FRET acceptor fluorophore can be added to the solution. FRET between donor and acceptor will report on the dynamic formation and dissociation of protein-RNA interactions. For a multi-color set-up, a second ligand (protein B) labeled with a different FRET acceptor dye (slightly red-shifted) allows to probe additional interactions between RNA and both proteins over time to study the assembly kinetics of a ternary complex [70]. (**B**) *In vivo*: a single-molecule RNA biosensor introduced to the cell via transfection of its corresponding plasmid DNA template allows monitoring the location of an mRNA molecule over time. Multimeric structural RNA motifs get recognized by fluorophore-conjugated proteins that bind to these elements. Simultaneously, one can also observe translation events throughout this time, in which fluorescent epitope binders recognize multiple peptide stretches along the nascent polypeptide chains emerging from one or multiple elongating ribosomes.

In TIRF microscopy the sample is illuminated with a highly inclined laser, such that the angle of incidence is above the critical angle ($\theta_{c}$), which results in total internal reflection (Figure 2A). This creates an evanescent field at the glass/sample interface that decays exponentially in intensity [70] with increasing distance from the interface. By reducing unwanted background from the bulk solution, the signal to noise ratio of the immobilized molecules of interest is increased. Multi-color single-molecule fluorescence microscopy experiments [94–97] in combination with alternative laser excitation or pulsed interleaved excitation [98–100] can be used to image a system with multiple fluorophores, giving the opportunity to study multiple different species and processes simultaneously.

Single-molecule TIRF microscopy can be used together with Förster resonance energy transfer (FRET). This allows the probing for interactions between two dye-conjugated entities that are close in space (2–8 nm range [101]). An excited FRET donor fluorophore can transfer energy to the FRET acceptor fluorophore via interaction of transition dipoles. Here, the appropriate choice of fluorophores plays a critical role. FRET pairs consist of a donor and an acceptor fluorophore with a significant overlap between the donor's emission and acceptor's absorption spectra [98,100]. The FRET efficiency scales inversely to the power of six of the donor-acceptor distance and therefore, serves as a spectroscopic distance ruler.

In combination with single-molecule FRET (smFRET) (see Box 1), these experiments provide information on the kinetics of mRNP complex assembly, conformational changes occurring within a molecule or between various components of a multi-component system and, if paired with time-resolved measurements, allows tracking the reaction dynamics of multi-step processes in real-time. In the following, we discuss a few selected examples to illustrate the potential of *in vitro* single-molecule experiments to study systems of dynamic mRNP complexes involved in translation repression.

Translation can be repressed by *cis-*acting elements, such as rG4 quadruplexes. Recognizing and resolving rG4 structures within UTRs is an important element in promoting or inhibiting translation of mRNAs that contain those elements and rely on G4 binding proteins for correctly adjusting translation initiation efficiency [93,102]. The DEAH-box family helicase DHX36/Rhau specifically recognizes these rG4 elements [103]. smFRET has proven to be useful to study how DHX36 engages with its RNA substrate providing key insights in how it binds rG4s in two distinct modes: (1) ATP-independent rG4 unfolding due to tight interaction with monomeric DHX36, and (2) ATP-dependent step-wise refolding, that is dependent on a 3′ single-stranded RNA overhang [104]. Experiments involving mutations on the protein-RNA interface, informed by structural studies [103], showed how specific residues are involved in rG4 unfolding and mRNP complex stability.

Addressing another important mechanism of mRNA translation repression, multi-color smFRET was used to study miRNA target search, specifically how the miRISC is able to efficiently recognize its mRNA target sites [68]. Chandradoss et al. [68] characterized the miRNA seed region and found that only a small segment of the seed (corresponding to the 5′ nucleotides 2–4 of the miRNA) is necessary for transient nucleation of the miRISC on target sites but that the entire seed sequence (5′ nucleotides 2–8) is required for stable interaction with the target mRNA. While they found that the dissociation rates are dependent on the number of matching nucleotides, the association rates are insensitive to the number of matching base pairs. Studying simultaneously two target sites separated by 15 nucleotides, shuttling of the miRISC between both target sites was observed in real-time. They concluded that lateral 1D diffusion increases bound lifetimes. Longer bound lifetimes are due to the movement of miRISC molecules along the mRNA, a process which kinetically competes with miRISC dissociation. They also showed that the 3′ end of the miRNA interacts with the PAZ domain to facilitate lateral diffusion by destabilizing the 3′ tail base pairing.

The importance of the PAZ domain in interacting with the 3′ end of miRNA was confirmed and further investigations also showed that hidden conformations of Ago2 play an important role in mRNA target recognition [105]. For instance, studying a central bulge in the TC (Ago2·miRNA·target-mRNA), present when miRNA and target-mRNA do not fully match, revealed that the PAZ domain and the 3′ end of the miRNA within the complex exhibit a conformation with low FRET efficiency. This conformation was assigned to the one poised for target-directed miRNA degradation supported in structural studies [69], but with a larger distance between the 3′ end of the miRNA and PAZ domain. This adapted conformation likely positions the miRNA more effectively for protection against cellular nucleases. Furthermore, testing unusual substrates such as pre-miRNA451, which is cleaved by hAgo2 itself [106], showcased conformational differences when compared with the TC with fully matched guide and target mRNA. In summary, miRNA target search employs many different modes to ensure fast and efficient recognition of its target mRNAs using a heterogeneous ensemble of conformations between the C- and N-terminal lobe of Ago2, that can accommodate different substrates.

During 4E-BP-dependent repression, 4E-BPs also exhibit a variety of conformations that modulate their repressive activities, which is governed by their phosphorylation status. In an effort to understand how conformational dynamics are linked to post-translational modifications, Dawson et al. [107] and Smyth et al. [108] were able to combine NMR with smFRET to study the dynamics of 4E-BP2 with eIF4E and to show how the phosphorylation status of the C-terminal IDR informs on the folded domain. The combinatorial approach of NMR and smFRET helped to understand how hyperphosphorylation fine-tunes the affinity of 4E-BP2 for eIF4E *in vitro*.

## *In vivo* single-molecule tracking

While *in vitro* single-molecule imaging allows studying simplified systems at the highest possible spatiotemporal resolution and in a well-defined and controllable manner, it lacks the cellular complexity. *In vivo* single-molecule imaging (see Box 2) allows monitoring the processes in a physiological context.

Box 2. ***In vivo* single-molecule imaging**

For *in vivo* single-molecule imaging, cells can be transfected with reporter mRNAs that encode necessary elements that report on localization and translation activity of the same mRNA molecule over time (Figure 2B). To monitor translation elongation of ribosomes on single mRNA molecules, multiple peptide repeats encoded in the open reading frame can be recognized by fluorescently labeled epitope binders, for example in the SunTag system [109,110]. An increasing fluorescence signal over time therefore reports on translation elongation of a single mRNA molecule. To simultaneously track the localization of the individual mRNA molecules, multiple copies of artificial RNA aptamers, such as RNA stem-loops or pseudoknots usually placed in the 3′ UTR, can act as recognition elements for fluorescently tagged proteins, thereby increasing the fluorescence signal sufficiently for detection and long-time mRNA location tracking. While *in vivo* single-molecule imaging allows the tracking of single mRNA molecules in living cells, they come with additional challenges, especially in terms of fluorescence labeling of target biomolecules. In contrast with *in vitro* experiments, for which single proteins or nucleic acids can be site-specifically labeled with a single small and bright organic dye, multiple copies of these reporters are needed to improve the signal to noise ratio of single biomolecules within a cell. Introducing long artificial sequence elements in an mRNA of interest can affect function, such as mRNA stability [111] and therefore needs to be verified for each experimental system.

Using *in cellulo* tracking of single RNA molecules provided novel insights into how miRNA-mediated translation repression is coupled to miRISC binding to its target mRNA, and how miRISC binding affects mRNA decay, inhibition of translation initiation and subcellular relocalization into processing bodies (PBs) [112,113]. The authors were able to show that the presence of miRNA recognition sites reduce the overall abundance of the respective mRNAs, confirming that destabilization and decay of mRNAs is due to miRISC. In addition to mRNA degradation, imaging over longer periods of time revealed that translation was also repressed by preventing initiation. Already translating ribosomes on Ago-bound mRNAs would finish in a run-off fashion, which was supported by a steadily decreasing fluorescent translation signal. This is consistent with other studies that showed how Ago2 binding inhibits translation initiation [114–116]. However, at low levels of Ago2, re-initiations are possible suggesting that this mechanism of repression is leaky, and ribosomes can escape. Altogether, *in vivo* imaging of single mRNAs demonstrated the functional heterogeneity between different mRNA molecules and within different cells, stressing the need for obtaining data with single-molecule and single cell information. In contrary to *in vitro* single molecule experiments, spatial context within a cellular environment is provided by *in vivo* imaging. For example, Cialek et al. [112] showed that Ago2 tethering is likely responsible for recruitment of silenced mRNAs to PBs, raising the question of how mRNA stability is regulated inside PBs. Using single-molecule imaging, it was also shown how miRISC associates to target mRNAs immediately after nuclear export of mRNAs and that it recognizes already translating mRNAs preferentially before they are poised for degradation [113].

Taken together, single-molecule approaches both *in vitro* and *in vivo* allow us to study dynamics of mRNPs and provide mechanistic information on multi-step assembly processes relevant to translation regulation. *In vivo* imaging studies are limited in the sense as they rely on artificial enhancement of fluorophore signal by using multiple copies of epitopes that can be recognized by fluorescent probes and the diffraction limit of light complicates experiments for obtaining kinetic information for dynamic multicomponent systems. This stresses the need for single-molecule imaging by smFRET experiments *in vitro* as a complementary approach. Combining single-molecule experiments with other structural and other biophysical methods both *in vitro* and *in vivo* [117] will provide further relevant information such that we can obtain an ever-increasing understanding on how dynamic biomolecular interactions drive cellular function.

**Perspective**

- Given that translation dysregulation is the cause of severe diseases, exploring the dynamics of translation regulation is of high importance to understand the specific mechanisms in translation repression and for allowing us to discover new sensitive targets for drug development.
- *In vitro* single-molecule fluorescence microscopy of reconstituted systems and single-molecule tracking in living cells will continue to provide important mechanistic insights into the regulation of translation repression.
- Multicolor single-molecule methods will be instrumental to understand how the different steps in translation and its regulation are functionally coupled to each other [33,118] and moving forward, how translation is coupled to other cellular processes [119,120].

## Abbreviations

ASD: autism-like disorder
eIF: eukaryotic initiation factor
FRET: Förster resonance energy transfer
mRNP: messenger ribonucleoprotein
PABP: poly(A) binding protein
PAZ: Piwi–Argonaute–Zwille
PBs: processing bodies
PIC: pre-initiation complex
RBP: RNA binding protein
smFRET: single-molecule FRET
TC: ternary complex
TIRF: total internal reflection fluorescence
tRNA: transfer RNA
UTR: untranslated region
